# Consequences of the Edge Effect in a Commercial Enzyme-Linked Immunosorbent Assay for the Diagnosis of Lyme Neuroborreliosis

**DOI:** 10.1128/JCM.03280-20

**Published:** 2021-07-19

**Authors:** Tamara van Gorkom, Gijs H. J. van Arkel, Willem Voet, Steven F. T. Thijsen, Kristin Kremer

**Affiliations:** a Centre for Infectious Diseases Research, Diagnostics and Laboratory Surveillance, Centre for Infectious Disease Control, National Institute for Public Health and the Environment (RIVM), Bilthoven, The Netherlands; b Department of Medical Microbiology and Immunology, Diakonessenhuis Hospital, The Netherlands; c Department of Neurology, Diakonessenhuis Hospital, Utrecht, The Netherlands; University of Tennessee at Knoxville

**Keywords:** intra-assay variation, edge effect, ELISA, index calculation, Lyme neuroborreliosis

## Abstract

The diagnosis of Lyme neuroborreliosis (LNB) is based on neurological symptoms, cerebrospinal fluid (CSF) pleocytosis, and intrathecally produced *Borrelia*-specific antibodies. In most cases, the presence of intrathecally produced *Borrelia*-specific antibodies is determined by using an enzyme-linked immunosorbent assay (ELISA). The edge effect is a known phenomenon in ELISAs and can negatively influence the assay reproducibility and repeatability, as well as index calculations of sample pairs which are tested in the same run. For LNB diagnostics, an index calculation is used for which the relative amounts of *Borrelia*-specific antibodies in CSF and serum are measured to calculate a CSF/serum quotient, which is needed to calculate the *Borrelia*-specific antibody index (AI). The presence of an edge effect in an ELISA used for LNB diagnostics may thus have implications. In this study, we investigated the intra-assay variation of the commercial Enzygnost Lyme link VlsE/IgG ELISA used for LNB diagnostics and showed the presence of an edge effect. Minor adaptations in the ELISA protocol decreased this effect. The adapted protocol was subsequently used to test 149 CSF-serum pairs of consecutive patients received in a routine diagnostic laboratory. By simulation, we showed that, if the standard protocol would have been used, then the edge effect for this study population could have resulted in 15 (10.1%) false-pathological and two (1.3%) false-normal *Borrelia*-specific IgG AIs. Thus, the observed edge effect can lead to inaccurate LNB diagnoses. Our study underlines that the edge effect should be investigated when ELISAs are implemented in routine diagnostics, as this phenomenon can occur in any ELISA.

## INTRODUCTION

Lyme borreliosis is a tick-borne disease. In the Netherlands, the incidence of Lyme borreliosis has quadrupled in the last 2 decades, with an estimated number of 25,500 cases of erythema migrans and 1,500 cases of disseminated disease in 2017 (https://www.rivm.nl/en/Documents_and_publications/Common_and_Present/Newsmessages/2018/Lyme_disease_cases_have_quadrupled). The annual incidence rate of Lyme neuroborreliosis (LNB), a manifestation of the nervous system, was 2.6 (95% confidence interval [CI], 2.4 to 2.8) per 100,000 Dutch inhabitants in 2010 (1). Clinical symptoms of LNB include radiculopathy, cranial neuropathy (often facial palsy), paresthesia, meningitis, and meningoencephalitis. As prompt treatment has proven effective, an accurate diagnosis of these severe forms of Lyme borreliosis is pivotal (2).

The laboratory diagnosis of LNB includes the detection of intrathecally produced *Borrelia*-specific antibodies. Intrathecal antibody production is established by the measurement of the number of *Borrelia*-specific antibodies in cerebrospinal fluid (CSF) and serum followed by an antibody index (AI) calculation as described by Reiber and Peter (3, 4). *Borrelia*-specific antibodies in CSF and serum should be measured in the same run to exclude interassay variation and, hence, ensure optimal accuracy. Across the testing plate used for antibody detection, variations can occur that are most pronounced at the edges of an ELISA plate. These variations are shown by a difference in the optical densities (OD) between inner (lower OD) and outer (higher OD) wells of an ELISA plate and are referred to as the “edge effect” (5–7). The presence of intra-assay variations such as the edge effect could potentially lead to false-positive or false-negative results and should therefore be investigated when ELISAs are used in routine diagnostics (6, 8, 9). In this study, we investigated the performance of a commercial ELISA used for the diagnosis of LNB and observed an edge effect. We analyzed the consequences of this effect on the AI calculation and subsequent impact on LNB diagnostics through simulation. We also show some minor adaptations to the protocol which can lower the well variation across the plate.

## MATERIALS AND METHODS

### Verification of the Enzygnost IgG ELISA.

In a first experiment, the intra-assay variation of the commercial Enzygnost Lyme link VlsE/IgG ELISA (Siemens Healthcare Diagnostics, Marburg, Germany), here referred to as the Enzygnost IgG ELISA, was investigated. The Enzygnost IgG ELISA consists of a 96-well plate holder harboring 12 strips, each consisting of 8 wells coated with a mixture of deactivated Borrelia afzelii antigens (PKo isolate) and recombinant VlsE from B. afzelii, Borrelia garinii, and Borrelia burgdorferi *sensu stricto* (10). In this experiment, a total of 4 test strips (32 wells) were used, which were placed in the first 4 columns of a 96-well plate holder. Thirty wells were tested with a single dilution (1:231) of the positive kit control (human serum with specific IgG to B. burgdorferi), which was subsequently divided over 30 wells. The remaining wells were tested with the negative kit control (human serum without antibodies to B. burgdorferi) (Table 1A).

**TABLE 1:**
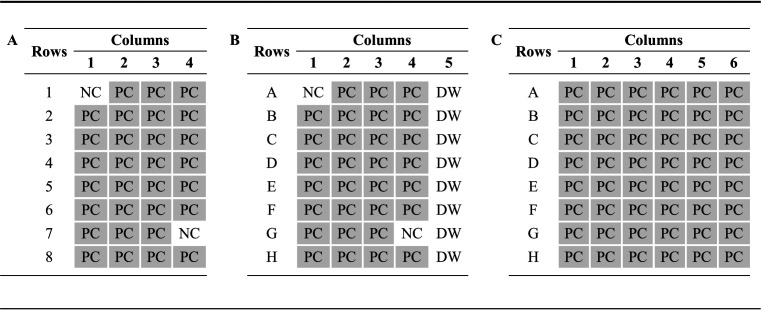
Overview of the setup of the three experiments performed in this study to investigate the intra-assay variation of the Enzygnost IgG ELISA^*a*^

a Panel A shows, in total, 32 wells that were tested in 4 strips, which were placed in the first 4 columns of a 96-well plate holder on an automated DS2 instrument (Dynex Technologies). A single dilution (1:231) of the positive kit control (PC) was tested in 30 wells, and the negative kit control (NC) was tested in 2 wells. Panel B is like panel A; however, an additional strip filled with distilled water (DW) was placed in the fifth column of the 96-well plate holder. In panel C, a single dilution (1:231) of the PC was tested in 6 strips (48 wells), which were placed in the first 6 columns of a 96-well plate holder on an automated BEP 2000 system (Siemens Healthcare Diagnostics).

In a second experiment, the intra-assay variation of the Enzygnost IgG ELISA was further investigated. The second experiment was identical to the first except for an additional strip filled with distilled water, which was placed in the fifth column of the 96-well plate holder (Table 1B). Both experiments were performed according to the instructions of the manufacturer by using a Dynex DS2 automated ELISA instrument (Dynex Technologies, Chantilly, VA, USA) (10).

In a third experiment, the intra-assay variation of the Enzygnost IgG ELISA was assessed by using a different ELISA robot. This experiment resembled the first; however, a single dilution (1:231) of the positive kit control was tested in 6 strips (48 wells) that were placed in the first 6 columns of a 96-well plate holder (Table 1C). The negative kit control was not included in this experiment. The ELISA was performed according to the instructions of the manufacturer by using the BEP 2000 system (Siemens Healthcare Diagnostics) (10).

### Intra-assay variation and statistical analyses.

To assess the intra-assay variation of the Enzygnost IgG ELISA, the intra-assay coefficients of variation (CV) of the OD values of the positive kit control were calculated by using the formula CV = (standard deviation of the OD values/mean OD value) × 100%. The CVs were calculated for each column, row, and plate. The negative kit control was not included in any of the analyses performed.

To further assess the performance of the assay, the median OD values of the columns were compared by using Friedman's test (11). Subsequent pairwise comparisons were done by using the Nemenyi *post hoc* test (https://CRAN.R-project.org/package=PMCMRplus). Raw *P* values of <0.05 were interpreted as statistically significant; however, to account for the multiple statistical analyses in this study, the Benjamini-Hochberg procedure (BH) was applied. Therefore, the false-discovery rate (FDR) was set at the level of 5.0%, and less than one false-positive result was allowed in the list of rejections (12). For all statistical analyses, RStudio (version 1.3.959, 2009 to 2020; Boston, MA, USA) was used (13). For construction of the figures, GraphPad Prism version 5.04 for Windows was used (GraphPad Software, San Diego, CA, USA).

### Standard versus adapted Enzygnost IgG ELISA protocol.

In the instruction manual of the manufacturer of the Enzygnost IgG ELISA, the following two standard protocols are described for the testing of CSF-serum pairs, which depends on the test system used: (i) for manual testing or testing with the automated BEP III system (Siemens Healthcare Diagnostics), each CSF-serum pair should be tested in consecutive wells, column-wise, starting with the serum sample; and (ii) for testing with the automated BEP 2000 system (Siemens Healthcare Diagnostics), all serum samples of the patients to be tested should be tested first in consecutive wells and column-wise, followed by all the corresponding CSF samples (14).

For use in our validation study (see next paragraph), the standard protocol was adapted in the following two ways before the patient samples were tested: (i) two strips filled with distilled water were added in each run, one located before the first strip, the other located after the last strip; and (ii) all CSF-serum pairs were tested row-wise in consecutive wells. For all other steps, the standard protocol was used (14).

### Criteria for the calculation of the *Borrelia*-specific CSF/serum IgG quotient and the *Borrelia*-specific IgG AI.

In order to obtain the CSF and serum concentrations needed to calculate the *Borrelia*-specific CSF/serum IgG quotient, we followed the instructions of the manufacturer (10). The raw OD values of the wells in each run were adjusted with a correction factor to correct for interassay variation. This correction factor was calculated for each run by using the following formula: correction factor = lot-specific OD value of the positive kit control/mean OD value of the positive kit control in each run. Subsequently, the corrected OD values were used to calculate the concentration (in units per milliliter) by using the α-method, however, only when the following two criteria were fulfilled: (i) the raw OD value should be 2.5 or lower, and (ii) the corrected OD value should be higher than that of the run-specific cutoff. This cutoff was based on the mean OD value of the negative kit control plus 0.150 (10).

According to the instructions of the manufacturer for LNB diagnostics, the *Borrelia*-specific CSF/serum IgG quotient may be calculated when the following two criteria are met: (i) the CSF and serum concentration must lie within the measurement range, and (ii) the concentration quotient must lie between 1.0 and 3.0 (14). The concentration quotient is calculated by dividing the higher concentration by the lower concentration before correction with the corresponding dilution factor. The lower concentration limit of the measurement range is run dependent and defined by the concentration of the run-specific cutoff. The upper limit of the measurement range is run independent and set at 300 U/ml. If the initial dilution of the CSF and/or serum results in a concentration that exceeds 300 U/ml, then further dilutions are needed before the *Borrelia*-specific CSF/serum IgG quotient may be calculated. In case multiple dilutions are tested, the CSF-serum pair that best fits the criteria for calculation of the *Borrelia*-specific CSF/serum IgG quotient is chosen. The *Borrelia*-specific CSF/serum IgG quotient is calculated by dividing the CSF concentration by the serum concentration after correction with the dilution factor. The *Borrelia*-specific IgG AI is calculated according to Reiber and Peter (4). AI values between 0.5 and 1.49 are considered normal, AI values ≥1.5 are considered pathological, and AI values <0.5 indicate a measurement error or a sample mix-up (14).

### Study population used to investigate the possible consequences of using the standard protocol for LNB diagnostics.

To investigate the possible consequences of using the standard protocol for the measurement of *Borrelia*-specific IgG in CSF-serum pairs and subsequent *Borrelia*-specific IgG AI calculation, we used the Enzygnost IgG ELISA results that were part of a larger validation study (T. van Gorkom, W. Voet, G. H. J. van Arkel, M. Heron, S. F. T. Thijsen, and K. Kremer, unpublished data). In this validation study, all consecutive patients were included from whom a CSF-serum pair, taken less than 24 h apart, was sent to the laboratory of the Diakonessenhuis Hospital, Utrecht, the Netherlands, in the period between August 2013 and June 2016. Until the start of this study in 2017, leftover material from these patients (both CSF and serum) had been stored at −20°C and/or −80°C. Prior to the start of this study, all samples had been freeze-thawed once to aliquot for use in multiple assays for the larger validation study and were stored at −20°C until use. Patients for whom an insufficient amount of CSF and/or serum was available were excluded. In total, 156 consecutive patients were included in this study, and the CSF and serum pairs of 149 of them were tested by using the Enzygnost IgG ELISA. Seventy-one (47.7%) of the 149 patients were male, and their mean age was 52.0 years (95% CI: 49.4 to 54.7). Twenty-nine (19.5%) patients had pleocytosis (≥5 leucocytes/μl) based on the CSF cell counts that had been determined at the time of the lumbar puncture in the past.

All CSF and serum pairs used in this study were anonymized and, according to the rules of our hospital, approval of the local ethics committee was not necessary as the main goal of our study was assay validation for which leftover material could be used. We did, however, obtain approval for this study from the hospital board.

### Construction of simulated Enzygnost IgG ELISA results by using two scenarios.

Intra-assay variation by using the standard protocol of the manufacturer can influence the ELISA results and, hence, the *Borrelia*-specific IgG AI. For instance, if the CSF of a CSF-serum pair is tested in a well located in a certain position in the plate that structurally leads to higher OD values than a well located elsewhere in the same plate, then this could result in a higher concentration, a higher *Borrelia*-specific CSF/serum IgG quotient, and a higher *Borrelia*-specific IgG AI. This could thus also affect the interpretation of the AI. To gain insight into the consequences thereof, we used the Enzygnost IgG results of the 149 consecutive patients that were obtained by using the adapted protocol. In this adapted protocol, CSF and serum of a CSF-serum pair were tested in such a way to minimize the intra-assay variation of the standard protocol of the manufacturer. By using these Enzygnost IgG results, we constructed so-called simulated Enzygnost IgG results that could have been obtained when the standard protocol would have been used, and either CSF or serum of a CSF-serum pair was tested in a well that was subjected to the intra-assay variation. To construct these simulated Enzygnost IgG ELISA results, the raw OD values of either the CSF or the serum of the CSF-serum pairs (obtained by using the adapted protocol) were subjected and adjusted to the intra-assay variation found in the first experiment. Therefore, we determined a factor that reflected the magnitude of the intra-assay variation. As the intra-assay variation was most prevalent between the columns in the first experiment, we based the factor on the largest difference in median (raw) OD value of the positive kit control found between any of the two columns. This factor was calculated by dividing the highest median OD value of any of the four columns by the lowest median OD value of any of the four columns. By using this factor, two scenarios were investigated. In the first scenario, the CSF of a CSF-serum pair was simulated as if it had been tested by using the standard protocol in a well in the column that displayed the highest median OD value. Therefore, the raw OD value of the CSF, obtained by using the adapted protocol, was adjusted through multiplication by the factor reflecting the magnitude of the intra-assay variation. In this scenario, the raw OD value of the serum, obtained by using the adapted protocol, was not adjusted. In the second scenario, the serum of a CSF-serum pair was simulated as if it had been tested by using the standard protocol in a well in the column that displayed the highest median OD value. Therefore, the raw OD value of the serum, obtained by using the adapted protocol, was multiplied by the factor reflecting the magnitude of the intra-assay variation. In this scenario, the raw OD value of the CSF, obtained by using the adapted protocol, was not adjusted. Subsequently, in both scenarios, the raw OD values were corrected, the concentrations were calculated, the simulated *Borrelia*-specific CSF/serum IgG quotients were determined, and, hence, the simulated *Borrelia*-specific IgG AIs were calculated according to the instructions of the manufacturer (and also described above) (10, 14). By using the simulated *Borrelia*-specific IgG AIs, the impact of the intra-assay variation was assessed.

## RESULTS

### The performance of the Enzygnost IgG ELISA based on the measured OD values.

To visualize the intra-assay variation of the Enzygnost IgG ELISA, heat maps of the OD values obtained in the first and second experiment were constructed, which showed that higher OD values were found in the outer wells than those in the inner wells (Table 2A and B). This phenomenon is also described as the edge effect (5–7). In the first experiment, an overall mean CV of 8.9% was found among the wells filled with the positive kit control. More variation was found between the OD values in the wells in rows than between the OD values in the wells in columns (mean CV, 8.4% and 4.0%, respectively; Table 2A). The addition of a strip filled with distilled water adjacent to the last test strip in the second experiment, thereby decreasing the number of outer wells, resulted in a lower overall mean CV of 4.4% (Table 2B). This also resulted in a lower variation between the OD values in the wells in rows, which was now comparable to the variation between the OD values in the wells in columns (mean CV, 3.2% and 3.8%, respectively; Table 2B).

**TABLE 2:**
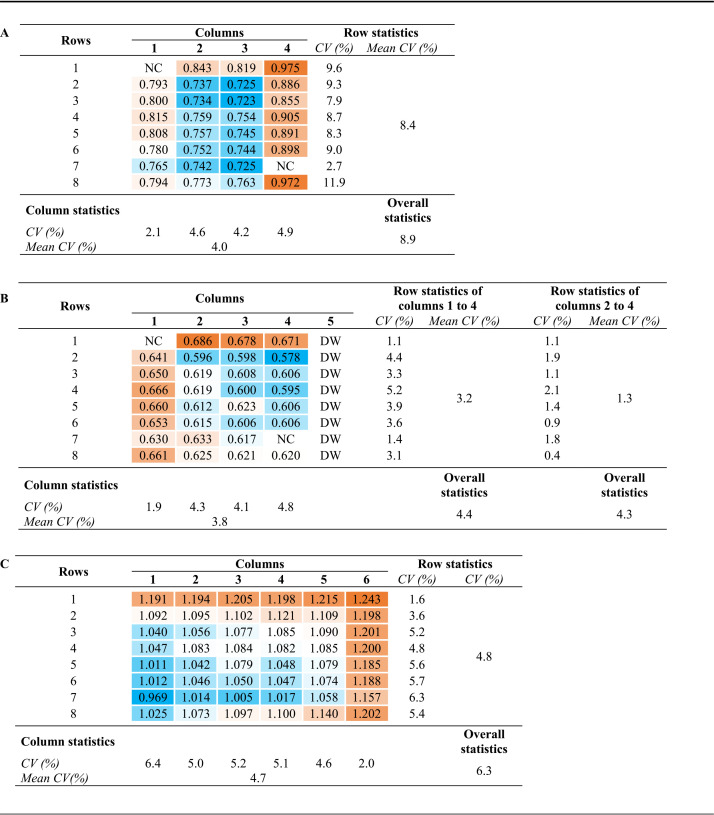
Heat maps and coefficients of variation (CV) of the optical density (OD) values of the positive kit control for the three experiments performed in this study^*a*^

a The constructed heat maps show the levels of the OD values of the positive kit control in all wells tested, from low (blue) to high (orange). Panel A shows a heat map and CV of the positive kit control of which a single dilution (1:231) was tested in 30 wells of 4 strips that were placed in the first 4 columns of a 96-well plate holder on an automated DS2 instrument (Dynex Technologies). Panel B is like panel A; however, an additional strip filled with distilled water was placed in the fifth column of the 96-well plate holder. Panel C is a heat map and CV of the positive kit control of which a single dilution (1:231) was tested in 48 wells of six strips that were placed in the first six columns of a 96-well plate holder on an automated BEP 2000 system (Siemens Healthcare Diagnostics). CV, coefficient of variation; NC, negative control; DW, distilled water.

**FIG 1:**
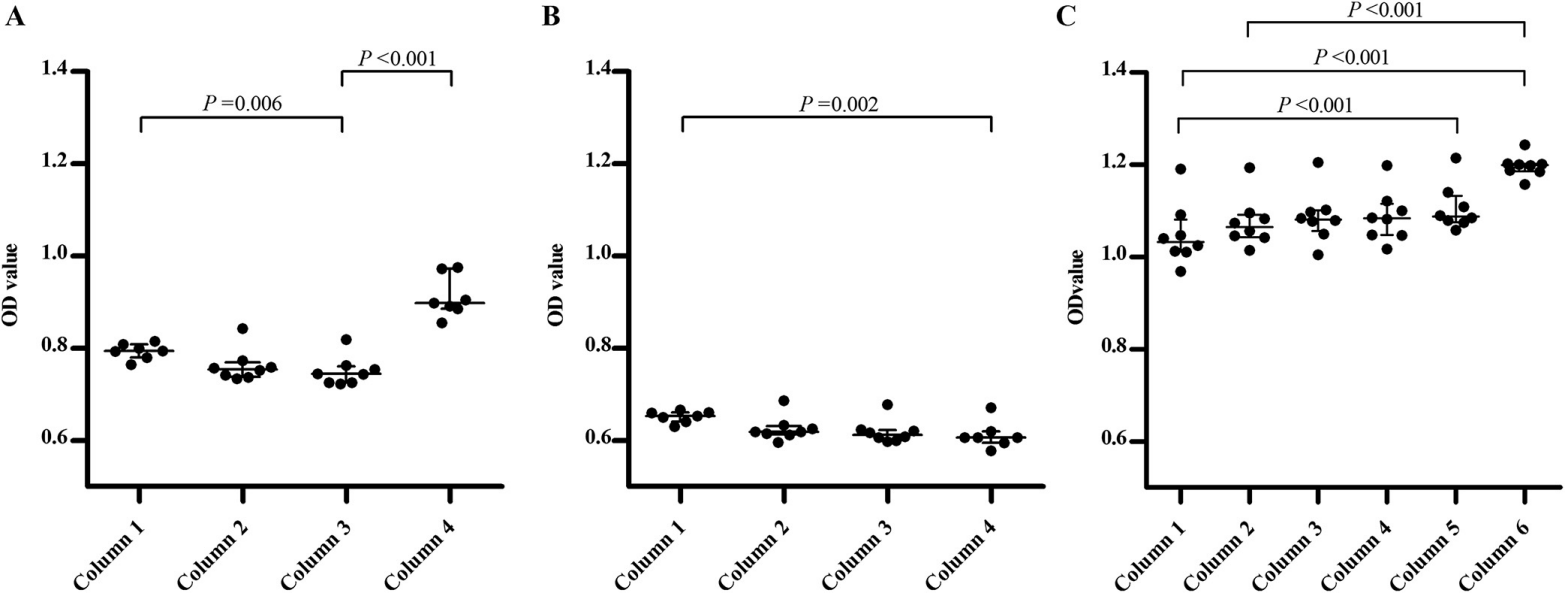
Scatter dot plots of the optical density (OD) values of the positive kit control in the three experiments. The horizontal lines indicate the median OD values, and the whiskers represent the interquartile ranges (IQR; first and third quartiles). The displayed *P* values are raw *P* values that were found to be significant after applying the Benjamini-Hochberg procedure to account for the multiple comparisons in this study. (A) Scatter dot plots of the OD values of the positive kit control of which a single dilution (1:231) was tested in 30 wells of 4 strips that were placed in the first 4 columns of a 96-well plate holder on an automated DS2 instrument (Dynex Technologies). (B) Like panel A, however, with an additional strip filled with distilled water placed in the fifth column of the 96-well plate holder. (C) Scatter dot plots of the OD values of the positive kit control of which a single dilution (1:231) was tested in 48 wells in 6 strips that were placed in the first 6 columns of a 96-well plate holder on an automated BEP 2000 system (Siemens Healthcare Diagnostics).

Further analyses showed that the OD values in the wells in the outer columns (columns 1 and 4 in the first experiment and column 1 in the second experiment) were higher than those in the wells in the inner columns (Table 2A and B; Fig. 1A and B). In the first experiment, the OD values in the wells in both the first and the fourth (both outer) columns were significantly higher than those in the third (inner) column (Fig. 1A). In the second experiment, the OD values in the wells in the first (outer) column were significantly higher than those in the fourth (now inner) column (Fig. 1B). By exclusion of the wells in the first column, only wells of similar location, either all located outside (first and last row) or all located inside (rows 2 to 7), were compared. This resulted in a further decrease of the variation found between the OD values in the wells in rows (mean CV, 1.3%) (Table 2B).

The third experiment was similar to the first experiment except for the use of a different automated ELISA instrument and the number of wells (48 instead of 30) filled with the positive kit control (Table 1C). Again, the heat map of the OD values showed that intra-assay variation was present across the wells tested (Table 2C). The variation was slightly different than that found in the first experiment. Still, an edge effect was observed, as higher OD values were found in the outer wells (sixth column and first row) than in the inner wells (Table 2C). In contrast to what was found in the first and second experiments, the OD values were lowest in the wells in the first column and increased from left to right (Fig. 1C). Thus, the OD values in the wells in the sixth column were the highest, which was significant compared to those in the first and second column (Fig. 1C). The OD values in the wells in the fifth column were also significantly higher than those in the first column.

### *Borrelia*-specific IgG AI results by using the adapted Enzygnost IgG ELISA protocol.

**FIG 2:**
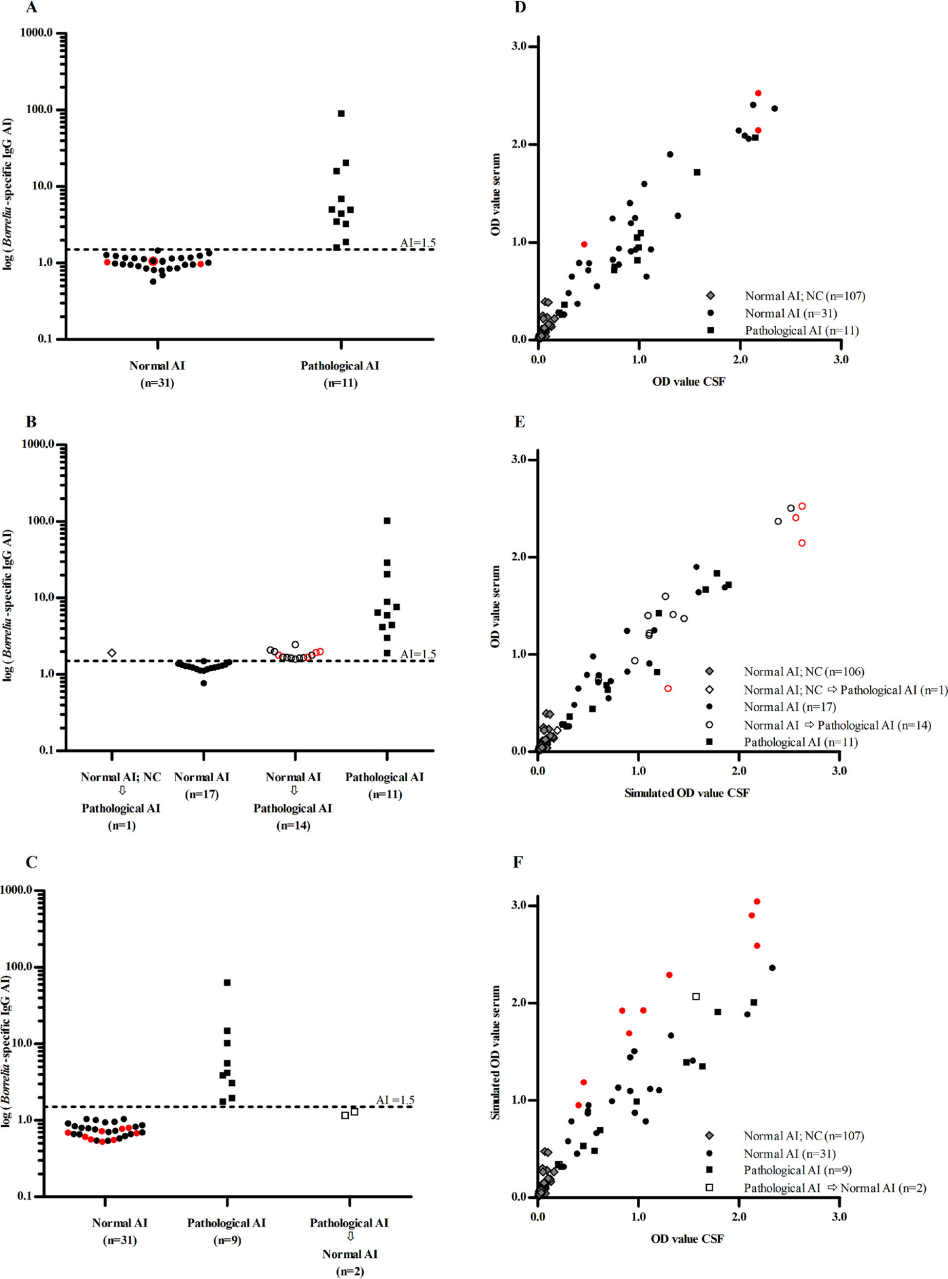
Scatter dot plots of the (log-transformed) *Borrelia*-specific IgG antibody indices (AI) obtained by using the adapted protocol (*n* = 42) (A) or the simulated standard protocol based on either the first (*n* = 43) (B) or the second (*n* = 42) (C) scenario and the XY-plots of the (corrected) optical density (OD) values of the 149 CSF-serum pairs obtained by using the adapted protocol (D) or the simulated standard protocol based on either the first (E) or the second (F) scenario. The adapted protocol shows the results obtained when both CSF and serum of a CSF-serum pair were tested in outer wells, or in inners wells, to minimize the edge effect. The simulated standard protocol shows the results obtained when either CSF or serum of a CSF-serum pair was subjected to the edge effect by investigating two scenarios. In the first scenario, the CSF was simulated to be tested in an outer well and the corresponding serum in an inner well. In the second scenario, the serum was simulated to be tested in an outer well and the corresponding CSF in an inner well. Simulation of testing in an outer well was done by multiplication of the raw OD value of either CSF (scenario 1) or serum (scenario 2) of a CSF-serum pair, obtained in the adapted protocol, with the edge-effect factor of 1.21 to reflect the magnitude of the edge effect. All results are grouped by the results of the *Borrelia*-specific IgG AI (i.e., a normal AI for AIs that were not calculated [NC] due to undetectable levels of *Borrelia*-specific IgG in the CSF, a normal AI for AI values between 0.5 and 1.49, and a pathological AI for AI values ≥1.5). Red symbols show the CSF-serum pairs that did not fulfill the criteria for calculation of the *Borrelia*-specific CSF/serum IgG quotient and, hence, the *Borrelia*-specific IgG AI, which, in practice, would not have been calculated (see also Table 3).

For LNB diagnostics, CSF and serum of a CSF-serum pair should be tested in the same run. In practice, by using the standard protocol of the manufacturer for the Enzygnost IgG ELISA, one of the two materials (either CSF or serum) could be tested in an outer well, and the other material could be tested in an inner well (14). Consequently, the CSF-serum pair could be subjected to the edge effect that was observed in the first experiment. To minimize the consequences of the edge effect, we adapted the protocol of the Enzygnost IgG ELISA for use in our larger validation study. In this adapted protocol, strips filled with distilled water were placed in the first and last columns of the run, and CSF and serum from a single patient were tested in consecutive wells in the same row, as less variation was seen between wells of similar origin (both inner or both outer wells) as was shown in the second experiment. In total, CSF-serum pairs from 149 patients were tested in the Enzygnost IgG ELISA by using the adapted protocol. For 138 (92.6%) of the 149 patients, no proof for intrathecal *Borrelia*-specific IgG production was established. For 107 (71.8%) of the 149 patients, no detectable levels of *Borrelia*-specific IgG in the CSF were found, and, therefore, no AIs were calculated (Table 3 and Fig. 2A). Of those 107 patients, 18 (16.8%) had an elevated CSF cell count (≥5 leucocytes/μl; data not shown).

**TABLE 3:** Comparison of the *Borrelia*-specific IgG AI results of the 149 CSF-serum pairs by using the Enzygnost IgG ELISA results obtained by using the adapted protocol and by simulating the standard protocol

Result	AI results (no. [% of total] for protocol:						
	Adapted protocol^*a*^	Simulated standard protocol^*b*^ in:					
		Scenario 1			Scenario 2		
		Normal, not calculated^*c*^	Normal, AI < 1.5	Pathological, AI ≥ 1.5	Normal, not calculated^*c*^	Normal, AI < 1.5	Pathological, AI ≥ 1.5
Normal, not calculated^*c*^	107 (71.8)	106		1	107		
Normal, 0.5 ≤ AI < 1.5	31^*d*^^,^^*e*^^,^^*f*^ (20.8)		17	14^*d*^^,^^*f*^^,^^*g*^^,^^*h*^		31^*d*^^,^^*e*^^,^^*f*^^,^^*h*^^,^^*i*^	
Pathological, AI ≥ 1.5	11 (7.4)			11		2	9
Total	149	106 (71.1)	17 (11.4)	26 (17.4)	107 (71.8)	33 (22.1)	9 (6.0)

a Adapted protocol shows the *Borrelia*-specific IgG AI results obtained when CSF and serum of a CSF-serum pair were tested in such a way that the edge effect was minimized (both outer or both inner wells).

b Simulated standard protocol shows the *Borrelia*-specific IgG AI results obtained when either CSF or serum of a CSF-serum pair was subjected to the edge effect by investigating two scenarios. In the first scenario, the CSF was simulated to be tested in an outer well and the corresponding serum in an inner well. In the second scenario, the serum was simulated to be tested in an outer well and the corresponding CSF in an inner well. Simulation of testing in an outer well was done by multiplication of the raw OD value of either CSF (scenario 1) or serum (scenario 2) of a CSF-serum pair, obtained in the adapted protocol, with the edge-effect factor of 1.21 to reflect the magnitude of the edge effect.

c Calculation of the AI was not possible due to the absence of *Borrelia*-specific antibodies in the CSF.

d For one CSF-serum pair, the antibody concentrations for CSF and serum exceeded the upper measurement limit of 300 U/ml in the adapted protocol (323 U/ml and 310 U/ml, respectively), in the first scenario (558 U/ml and 310 U/ml, respectively), and in the second scenario (323 U/ml and 534 U/ml, respectively).

e For one CSF-serum pair, the concentration quotient exceeded the upper concentration quotient limit of 3.0 in the standard protocol and in the second scenario (3.1 and 4.4, respectively; serum concentration > CSF concentration for both).

f For one CSF-serum pair, the antibody concentration of the serum exceeded the upper measurement limit of >300 U/ml in the adapted protocol and in the first and second scenarios (302 U/ml, 302 U/ml, and 525 U/ml, respectively), and that of the CSF exceeded the upper measurement limit in the first scenario (339 U/ml).

g For one CSF-serum pair, the concentration quotient in the first scenario was 3.3 (CSF concentration > serum concentration) and exceeded the upper concentration quotient limit of 3.0.

h For one CSF-serum pair, the antibody concentration of the CSF in the first scenario (316 U/ml) exceeded the upper measurement limit of 300 U/ml. In the second scenario, the antibody concentration of the serum (454 U/ml) exceeded this limit.

i For five CSF-serum pairs, the concentration quotients (range, 3.4 to 5.4; serum concentration > CSF concentration for all) exceeded the upper concentration quotient limit of 3.0.

For 42 (28.2%) of the 149 patients, an AI was calculated. The majority of them (31/42, 73.8%) had a normal *Borrelia*-specific IgG AI (Table 3 and Fig. 2A) of whom five (16.1%) had an elevated CSF cell count (≥5 leucocytes/μl; data not shown). Three (9.7%) of the 31 cases did not fulfill the criteria defined by the manufacturer required to calculate the *Borrelia*-specific CSF-serum IgG quotient and should have been repeated in a next ELISA run by using different dilutions (Table 3). For 11 (7.4%) of the 149 cases, a pathological *Borrelia*-specific IgG AI was found and thus proof of intrathecally produced *Borrelia*-specific IgG (Table 3 and Fig. 2A), and 6 (54.5%) of them had an elevated CSF cell count (≥5 leucocytes/μl; data not shown).

### Consequences of the observed edge effect in the Enzygnost IgG ELISA on the *Borrelia*-specific IgG AI determined by simulating the Enzygnost IgG ELISA results as if the standard protocol had been used.

By using the standard protocol, patient samples could be subjected to the edge effect. The magnitude of this edge effect was expressed by a factor which was calculated by dividing the highest median OD value (0.898; column 4) by the lowest median OD value (0.745; column 3) (Table 2A). This resulted in an “edge-effect factor” of 1.21, e.g., the OD values in the fourth column were 1.21 times higher than those in the third column. The presence of an edge effect could potentially lead to a false-pathological AI for those cases in which the CSF is tested in an outer well and the corresponding serum is tested in an inner well, as this results in a higher CSF/serum quotient and, thus, a higher AI. In contrast, a false-normal AI could be found when the serum is tested in an outer well and the corresponding CSF is tested in an inner well, as this will result in a lower CSF/serum quotient and, hence, a lower AI. Therefore, the edge-effect factor was used to create simulated Enzygnost IgG ELISA results in order to provide insight into the possible consequences for LNB diagnostics in case the standard protocol had been used. Two scenarios were investigated.

In the first scenario, in which the raw OD value of the CSF of a CSF-serum pair was multiplied by the edge-effect factor of 1.21, the number of *Borrelia*-specific IgG AI values greater than or equal to 1.5 increased from 11 to 26 (Table 3 and Fig. 2B). Thus, 15 CSF-serum pairs changed from a normal to a pathological *Borrelia*-specific IgG AI and were considered false pathological. All false-pathological AI values were located just above the AI cutoff value of 1.5 (Fig. 2B). For two (13.3%) of the 15 patients with a false-pathological AI, the CSF cell count was elevated (≥5 leucocytes/μL; data not shown). For four patients, all of whom had a false-pathological AI, the criteria for calculation of the *Borrelia*-specific CSF/serum IgG quotient were not fulfilled. Ideally, these CSF-serum pairs should be repeated in a next ELISA run by using different dilutions (Table 3).

In the second scenario, in which the raw OD value of the serum of a CSF-serum pair was multiplied by the edge-effect factor of 1.21, the number of *Borrelia*-specific IgG AI values greater than or equal to 1.5 decreased from 11 to 9 (Table 3 and Fig. 2C). Therefore, the results for two patients were considered to be false normal. Both false-normal AI values were located just below the AI cutoff value of 1.5 (Fig. 2C), and for both CSF samples, a normal cell count (<5 leucocytes/μl) was found (data not shown). By using the second scenario, the criteria for calculation of the *Borrelia*-specific CSF/serum IgG quotient were not fulfilled for nine patients (all of whom had a normal AI). Ideally, these CSF-serum pairs should be repeated in a next ELISA run by using different dilutions (Table 3).

### Analyses of the corrected OD values among the 149 CSF-serum pairs obtained by using the adapted protocol and those obtained in the two simulated scenarios.

The *Borrelia*-specific IgG AI does not provide insight into the OD values of the CSF and serum used to calculate the *Borrelia*-specific CSF/serum IgG quotient and the *Borrelia*-specific IgG AI. Yet this information is important for the interpretation of the AI, as a pathological AI can result from low (or high) OD values and might not indicate disease. The relationship between the OD values (plotted on the *y* axis) and the (log-transformed) concentrations (plotted on the *x* axis) is described by a sigmoid-shaped curve. When the OD values of both materials are either located in the lower left corner or the upper right corner of the curve, small differences between the OD values will result in larger differences between the concentrations, as is reflected by an almost horizontal line in the sigmoid-shaped curve in both corners. Thus, when the OD value of the serum is located in the lower left part of the curve on the horizontal line (and is lower than the OD value of the CSF), or when the OD value of the CSF is located in the upper right part of the curve on the horizontal line (and is higher than the OD value of the serum), the difference in concentration will be larger, which will result in an increased CSF/serum quotient as well as an increased AI. Therefore, AIs should be interpreted with care.

The XY-plots that were constructed for each of the three protocols (i.e., the adapted protocol and the two scenarios used to simulate the standard protocol) show the distribution of the OD values of the 149 CSF-serum pairs (Fig. 2D to F). In each protocol, the CSF-serum pair was selected that best matched the criteria for calculation of the *Borrelia*-specific CSF/serum IgG quotient. Consequently, for 17 (40.5%) of the 42 patients for whom an AI was calculated by using the adapted protocol, a different set of dilutions was used in at least one of the two scenarios in which the standard protocol was simulated (data not shown). Analyses of the XY-plots showed that the distribution of the OD values of CSF and serum used for the index calculation were comparable between the three protocols (Fig. 2D to F). For those cases for which an AI had not been calculated in any of the three protocols, the OD values of CSF and serum were located in the lower left corner of the plots (Fig. 2D to 2F). The OD values of the CSF and serum for those patients for whom the calculated AI was normal were distributed across the whole range of OD values for both CSF and serum, irrespective of the protocol used (Fig. 2D to F). Similarly, the OD values of the patients who had a pathological AI were distributed across the whole range of OD values for both CSF and serum and covered the same OD range as those who had a normal AI. For the 14 patients with a normal AI when using the adapted protocol and a false-pathological AI in the first scenario, the OD values were also distributed across the whole range of OD values for both CSF and serum. For a single patient for whom the AI was not calculated by using the adapted protocol and in the second scenario, and who had a pathological AI in the first scenario, the OD values were relatively low (Fig. 2E).

## DISCUSSION

The commercial Enzygnost Lyme Link VlsE/IgG ELISA can be used for the diagnosis of LNB by the detection of *Borrelia*-specific IgG in CSF-serum pairs (10, 14). For LNB diagnostics, the results of the CSF-serum pairs are used to calculate a *Borrelia*-specific CSF/serum IgG quotient, after which a *Borrelia*-specific IgG AI is calculated according to Reiber and Peter (4). The reliability of the ELISA results, however, can be affected by variations across the plate. In the current study, an edge effect was established for the Enzygnost IgG ELISA when run on a Dynex DS2 automated ELISA instrument (Dynex Technologies). The edge effect contributed to an overall mean CV of 8.9% in the OD values of a single dilution of the positive kit control. The edge effect was decreased by lowering the number of outer wells, which resulted in an overall mean CV of 4.3%. Similar results were obtained when the Enzygnost IgG ELISA was run on an automated BEP 2000 ELISA system (Siemens Healthcare Diagnostics).

In this study, we showed that the impact of the edge effect on CSF-serum pairs could be reduced by some minor adaptations to the standard protocol of the manufacturer (14), which, in this case, only applies for testing on the Dynex DS2 automated ELISA instrument (Dynex Technologies). For use in LNB diagnostics, the standard protocol of the Enzygnost IgG ELISA prescribes that CSF-serum pairs should be tested column-wise, either in consecutive wells or in wells placed farther apart. Consequently, one of the two materials could be located in an outer well and be subjected to the edge effect. In this study, we showed that when CSF-serum pairs are tested in wells of the same row (either both inside or both outside), less intra-assay variation is found. To ensure that only inner columns are used, a strip filled with distilled water should be placed before the first test strip and after the last test strip in a single run. Although we only tested the effect of placing a strip of distilled water in the last column, which can be seen as a limitation of this study, we believe the same effect will occur when a strip filled with distilled water is placed in the first column, as was shown in the second experiment. In this experiment, less variation was seen by the removal of the OD values obtained in the wells in the first column. The only difference is that the wells in the first column were filled with the positive kit control instead of distilled water; however, we do not believe this has had an impact. We also did not test the use of a strip filled with distilled water in the top row, which also consisted of outer wells for which higher OD values were obtained than those in the other rows. Yet, as variation between the wells in the top row was low (mean CV of 1.1% in the second experiment) and the first and last well were not used, as they were filled with distilled water, this row can be used in index calculations as long as both samples are tested in wells in this row. Another limitation of our study was the inclusion of three CSF-serum pairs for which the criteria of the manufacturer for the calculation of the *Borrelia*-specific CSF/serum IgG quotient and the *Borrelia*-specific IgG AI were not fulfilled by using the adapted protocol. Ideally, these should have been repeated; however, as the measurements barely fell outside the manufacturers’ criteria, we decided to use these results in the study. Moreover, we argue that these minor transgressions would probably be accepted and reported in routine clinical/laboratory practice. By simulating the standard protocol, the criteria for the calculation of the *Borrelia*-specific CSF/serum IgG quotient and the *Borrelia*-specific IgG AI were not met for 4 out of the 149 (for whom the CSF sample was simulated to be tested in an outer well) and for 9 out of 149 (for whom the serum was simulated to be tested in an outer well) CSF-serum pairs. In practice, by using the standard protocol, more dilutions would have been needed to obtain valid results for these cases.

The edge effect found in this study could be caused by differences in temperature across the plate, with lower temperatures in the center of the plate than in the edges, as has been described previously (5, 7, 15). Increased temperatures at the edges will cause more evaporation in outer wells and results in higher OD values (16, 17). Also, wells located at the edges are not fully surrounded by other wells, which can affect the temperature in those wells (15), and temperature differences between filled wells (higher) and empty wells (lower) have also been described (5). Variations across the plate could also be caused by differences in surface properties or unequal protein binding (8, 15, 18).

We demonstrated that the observed edge effect found in the Enzygnost IgG ELISA could have an impact on LNB diagnostics, especially when CSF is tested in an outer well and serum in an inner well. The magnitude of this impact was shown by calculating simulated Enzygnost IgG ELISA results by using the results of 149 CSF-serum pairs that were part of a larger validation study (T. van Gorkom, W. Voet, G. H. J. van Arkel, M. Heron, S. F. T. Thijsen, and K. Kremer, unpublished data). Depending on which sample (either CSF or serum) was placed in an outer well, either 15 (10.1%) of the 149 CSF-serum pairs resulted in a false-pathological AI, or 2 (1.3%) resulted in a false-normal AI. The number of false-pathological and false-normal results obtained through simulation was based on a worst-case scenario. That is, the edge-effect factor that was used to calculate the simulated CSF or serum results was based on selecting the two columns for which the difference between the median OD values was largest. Subsequently, these simulated CSF or serum results were used to calculate the simulated *Borrelia-*specific CSF/serum IgG quotients and, hence, the simulated *Borrelia*-specific IgG AIs. All CSF results, or all serum results, were subjugated to this simulated worst-case edge effect. Both the false-pathological AI values and the false-normal AI values were located close to the AI cutoff value of 1.5. This value, which is established by the manufacturer, is most likely based on the use of wells that perform similarly. Yet our study demonstrates that the AI cutoff value is too low for those cases in which the CSF is subjected to the edge effect (i.e., tested in an outer well in the standard protocol) and the serum is not (i.e., tested in an inner well in the standard protocol). Likewise, the AI cutoff value is too high for those cases in which the serum is subjected to the edge effect (i.e., tested in an outer well in the standard protocol) and the CSF is not (i.e., tested in an inner well in the standard protocol).

Our study suggests that by using the adapted protocol, false-pathological and false-normal AIs caused by the edge effect can be avoided. A study of Wang and Cheng, in which the concentration of the monoclonal antibody bevacizumab was measured in plasma of beagle dogs by using a similar platform (ELISA), also demonstrated an edge effect, and they showed that exclusion of the wells at the edges decreased this effect (9). Our study underlines the importance of investigating the performance of a new ELISA before it is implemented in routine diagnostics, as is required, e.g., by the ISO 15189 accreditation (19), as plate variation may apply to other ELISAs as well and may also depend on the test system used.
